# TLR4 deletion reduces small fiber sensory abnormalities and nerve degeneration in diabetic male mice

**DOI:** 10.1016/j.neuroscience.2025.10.052

**Published:** 2025-10-28

**Authors:** Sarah Crowards, Janelle Ryals, Lana Heslop, Will Hauser, Gentry Totta-Griese, Trent Madden, Douglas E. Wright

**Affiliations:** Department of Anesthesiology, University of Kansas Medical Center, Kansas City, KS 66160, United States

**Keywords:** Diabetes, Neuropathy, Inflammation, TLR4, Sensation, Pain

## Abstract

Diabetic peripheral neuropathy (DPN) is a prevalent complication of diabetes, significantly impairing quality of life and lacking effective disease-modifying treatments. Chronic inflammation involving toll-like receptor 4 (TLR4) has been implicated in diabetes and neuropathy development. TLR4 is an innate immune receptor that triggers an inflammatory response, leading to the production of pro-inflammatory cytokines. While TLR4 has been studied in various neuropathy models, its role in diabetic neuropathy and its effect on nerve fiber integrity remains unclear. To investigate the impact of TLR4 on DPN, we induced diabetes in wild-type and TLR4 knockout (TLR4−/−) mice using streptozotocin. Groups contained 8–18 animals and were approximately half male and half female. Over six weeks, we assessed blood glucose, weight changes, thermal and mechanical sensory function, hind paw intraepidermal nerve fiber density (IENFD), dermal macrophage accumulation, and serum cytokines. In males, TLR4 deletion protected against STZ-induced thermal hyposensitivity, decreases in IENFD, and dermal macrophage accumulation. Female mice developed less severe hyperglycemia and were resistant to neuropathic changes, making the protective effects of TLR4 deletion less pronounced in females than in males. Our findings confirm that TLR4 plays a role in DPN pathogenesis in a diabetic mouse model, demonstrating that its deletion promotes sensory function and preserves IENFD in males. These results highlight TLR4 as a potential therapeutic target for slowing the progression of neuropathy in diabetes. Our data also emphasizes the need for further research into the role of sex-specific disease mechanisms in DPN.

## Introduction

Diabetic peripheral neuropathy (DPN) is a common complication of diabetes, affecting up to 50 % of diabetic patients and typically presenting in a stocking-glove pattern with numbness, tingling, and burning pain (Feldman et al., 2019). DPN significantly reduces quality of life compared to diabetes alone, impairing energy, mobility, and sleep (Benbow et al., 1998). The International Diabetes Federation estimates that 1 in 8 adults will have diabetes by 2045, further increasing the prevalence of DPN (Diabetes Atlas, 2021). This also poses a substantial financial burden on the healthcare system, with a median cost of approximately $9,000 per diabetic patient with nonpainful neuropathy in the first year after diagnosis, and nearly double that in those with painful neuropathy (Kiyani et al., 2020). Despite its impact, treatment options remain limited, consisting of three oral medications (pregabalin, duloxetine, and tapentadol), one topical agent (capsaicin 8 %), and spinal cord stimulation devices (Mallick-Searle & Adler, 2024). These treatments manage symptoms but do not prevent disease progression, underscoring the need for a deeper understanding of the pathophysiology of DPN to develop more effective therapies.

Chronic low-grade inflammation has been observed in both type 1 and 2 diabetes and is thought to contribute to their complications, including DPN (Pop-Busui et al., 2016). Components of the innate immune system, such as macrophages, inflammatory cytokines, and toll-like receptors (TLRs), play a role in disease progression by promoting tissue damage, oxidative stress, and endothelial dysfunction (Banu & Sur, 2023; Espinoza-Jiménez et al., 2012; Yang et al., 2009; Yehualashet, 2020; Zheng et al., 2021). TLR4, in particular, has emerged as a receptor of interest in neuropathy and diabetes research. Classically activated by lipopolysaccharide (LPS), TLR4 signaling triggers the expression of pro-inflammatory genes, including cytokines such as TNF and IL-6 (Sameer & Nissar, 2021). Elevated TLR4 expression has been observed in individuals with type 1 and type 2 diabetes (Devaraj et al., 2008; Zhu et al., 2015), alongside increased levels of known TLR4 agonists, including gut-derived LPS, in their serum (Aravindhan et al., 2015; Devaraj et al., 2009; Jayashree et al., 2014; Rogero & Calder, 2018). In fact, TLR4 mRNA and protein expression are significantly higher in patients with DPN compared to those with type 2 diabetes alone and may serve as sensitive diagnostic markers for DPN (Zhu et al., 2015). Notably, a single clinical study suggests that patients with painful DPN experienced symptom relief when treated with low-dose naltrexone, an inhibitor of TLR4 (Srinivasan et al., 2021).

Preclinical research in rodents also supports the role of TLR4 in the development of neuropathic sensory symptoms and the progression of diabetes. TLR2/4 knock-out mice fed a high-fat diet develop metabolic syndrome-related sensory neuropathy more slowly than wild-type controls (Elzinga et al., 2019). Additionally, TLR4 inhibition improves mechanical allodynia and thermal hyperalgesia and reduces immune cell infiltration into the dorsal root ganglion in a chronic nerve constriction model (Bettoni et al., 2008; Liu et al., 2016; Wu et al., 2010). Null mutations in TLR4 have been investigated in the context of several diabetic complications. In STZ-induced models of diabetes, TLR4-deficient mice are protected against nephropathy, renal inflammation, and podocytopathy (Jialal et al., 2014; Ma et al., 2014). TLR4 knock-out also confers protection against diabetic retinopathy, partially protects against diabetic bladder dysfunction, improves wound healing, and mitigates osteoporosis (Cao et al., 2021; Dasu & Jialal, 2013; Fu & Liu, 2023; Szasz et al., 2016). Additionally, TLR4-deficient NOD mice exhibit reduced lipid accumulation in cardiac muscle, potentially protecting against heart disease (Dong et al., 2012). While TLR4 inhibition has been shown to alleviate various outcomes of diabetes, its specific effects on neuropathy, including sensory changes and nerve fiber integrity, have not been directly studied.

Building on these findings, our study tested whether global TLR4 gene deletion, which has shown benefits in non-hyperglycemic neuropathy models, protects against diabetes-induced neuropathy. Our study also addresses epidermal nerve fiber integrity, a key diagnostic marker of DPN, which has not been assessed in previous neuropathy models associated with TLR4 deletion. We used a single high-dose STZ model, which more closely mimics the pancreatic insulin dysfunction found in type 1 diabetes. In this model, STZ accumulates in pancreatic β-cells, causing DNA double-strand breaks and impairing glucose transport (O’Brien et al., 2014). This results in the rapid onset of hyperglycemia, weight loss, and development of a neuropathic phenotype. Using this model in wild-type and TLR4 knockout (TLR4−/−) mice, we measured blood glucose levels, body weight, sensory function, intraepidermal nerve fiber density (IENFD), and dermal macrophage accumulation over a six-week period after diabetes induction. Our results demonstrate that TLR4 deletion protects against thermal hyposensitivity, nerve fiber loss, and macrophage infiltration in male mice, supporting a role for TLR4 in the progression of diabetic neuropathy.

## Materials and methods

### Animals

**Fig. 1.**
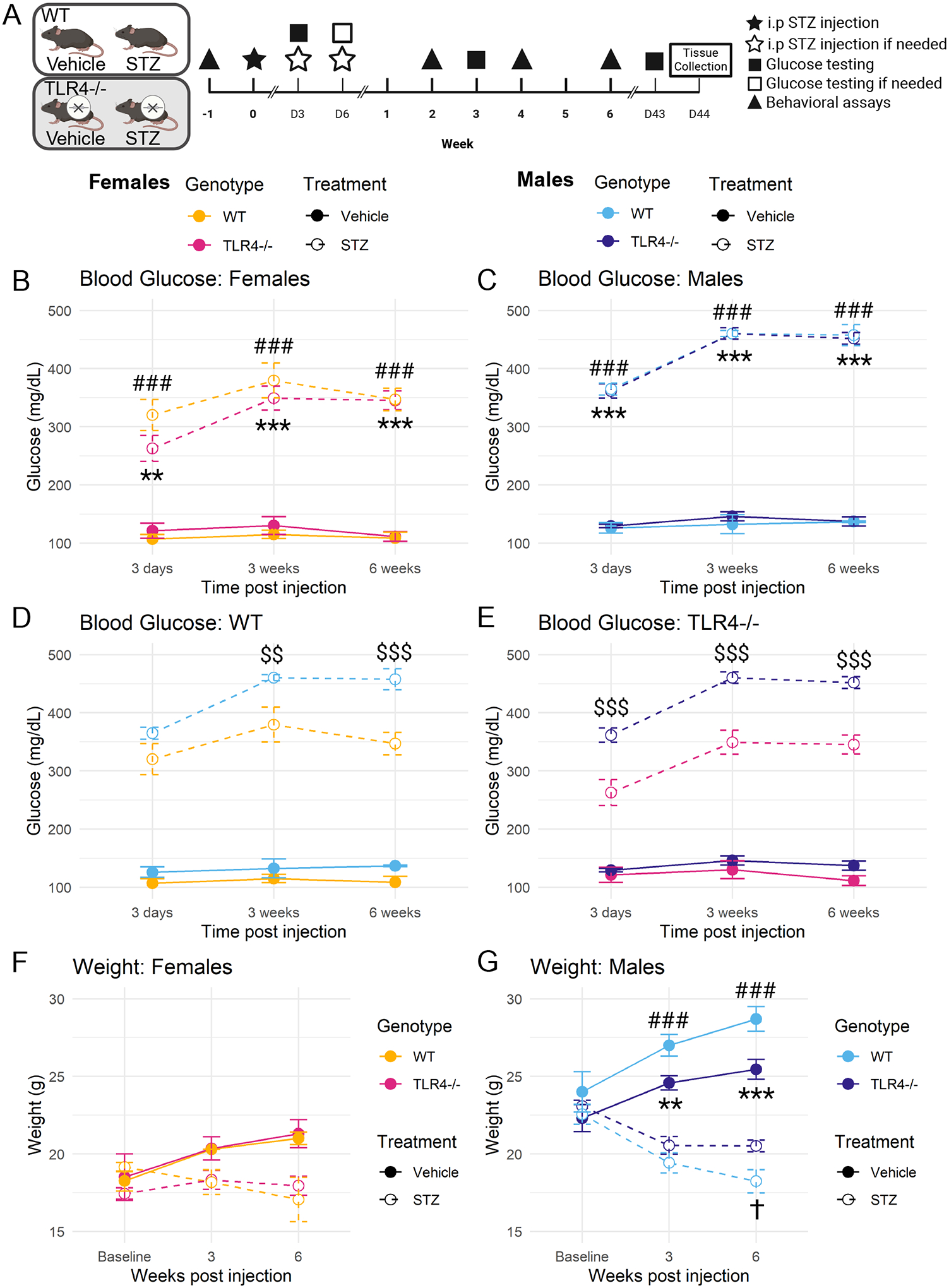
Experiment groups, timeline, and metrics of diabetes progression. (A) Visual representation of the experimental groups and time course carried out in C57BL/6J wild-type (WT) and TLR4−/− mice with and without STZ treatment. Blood glucose measured at 3 days (following either the initial or second dose of STZ, if required), and at 3 and 6 weeks after initial injection in STZ- or vehicle-treated WT and TLR4−/− female (B) and male (C) mice, as well as in female and male WT (D) and TLR4−/− (E) mice. Body weight at baseline, 3, and 6 weeks in STZ- or vehicle-treated WT and TLR4−/− female (F) and male (G) mice. Analyzed using one-way ANOVA at each time-point with Tukey’s HSD post-hoc test. ### p ≤ 0.001 STZ-treated WT vs vehicle-treated WT. ** p ≤ 0.01, *** p ≤ 0.001 STZ-treated TLR4−/− vs vehicle-treated TLR4−/−. $ $ p ≤ 0.01, $ $ $ p ≤ 0.001 STZ-treated male vs STZ-treated female. † p ≤ 0.05 STZ-treated WT vs STZ-treated TLR4−/−. n = 4–10. Data are represented as means ± SEM.

All animal work was performed following review and approval by the University of Kansas Medical Center Institutional Animal Care and Use Committee. Null mutant TLR4−/− mice were purchased from the Jackson Laboratory (Strain #:029015), and a colony was maintained in the animal research facility at the University of Kansas Medical Center. C57BL/6J mice were purchased from the Jackson Laboratory (Strain #:000664) as wild-type controls. Mice were 8–10 weeks old at the initiation of the experiment. All mice were maintained on a 12:12 light: dark cycle and given free access to water and standard rodent chow (TD.8604; Envigo; 14 % fat, 32 % protein, and 54 % carbohydrate by kcal). Wild-type and TLR4−/− mice were separated into STZ-injected and vehicle-injected groups. Mice were assigned to groups after completing baseline behavior to ensure that all groups had roughly equal baseline thermal and mechanical thresholds. After identifying and excluding mice that did not become diabetic as defined by a blood glucose ≥ 240 mg/dL (n = 9) or were euthanized early due to reaching a humane endpoint (n = 3), each group contained 8 to 18 animals and was approximately half male and half female (Table 1). Exclusion criteria were defined a priori. Sample size was chosen based on our experience that a certain percentage of mice would fail to develop STZ-induced diabetes and previous work in the lab defining the number of mice needed to provide sufficient power to detect behavioral differences. Investigators were blinded to all group assignments during behavioral and outcome assessments. Although they were blinded, phenotypic differences between STZ and vehicle-treated mice are apparent during behavioral analysis. To combat this, the investigator conducting behavioral assessments was blinded to mouse genotype. No obvious phenotypic differences exist between wild-type and TLR4−/− mice. A visual representation of the experimental groups and timeline is shown in Fig. 1A.

### Diabetes induction

Diabetes was induced by intraperitoneal (i.p.) injection of STZ (Sigma, Cat #S0130). Mice were fasted for three hours before and after receiving an i.p. injection of either 180 mg/kg STZ dissolved in sodium citrate buffer (pH 4.5) or control sodium citrate buffer. Blood glucose was measured at 72 h post-STZ injection to confirm induction of diabetes (blood glucose ≥ 240 mg/dL). Mice that did not develop hyperglycemia were given subsequent 30 mg/kg STZ injections and reevaluated by blood glucose every 72 h until they became diabetic or received a cumulative dose of 240 mg/kg STZ. A total of eight mice (wild-type: 2 females, 1 male; TLR4−/−: 4 females, 1 male) required one subsequent injection, and a total of four mice (wild-type: 1 female; TLR4−/−: 3 females) required two subsequent injections.

### Blood glucose measurements

Mice were fasted for three hours before drawing 20 μL of blood from the tail vein by clipping off the tail tip, or the resulting scab in subsequent blood draws. All standards and samples were mixed with molecular H_2_O, ZnSO_4_ (Sigma), and Ba(OH)_2_ (Sigma), then centrifuged at 14000 rpm at 4 °C for five minutes. The resultant supernatant was incubated for 30 min at 37 °C with color reagent (PGO capsule, Sigma; o-Dianisidine Dihydrochloride, Sigma; in molecular water). A plate reader (SpectraMax M5) was used to measure the absorbance of standards and samples in duplicate at 450 nm. Persistent hyperglycemia is generally accepted as the standard for successful diabetes induction in STZ models (Cao et al., 2021; Devaraj et al., 2011; Jialal et al., 2014; Ma et al., 2014). Additional measures, such as HbA1c and glucose tolerance, while also desirable in confirming diabetes, were not appropriate in this model due to the length of disease and invasiveness of testing.

### Sensory behavior testing

Sensory behavior testing was performed at baseline, two, four, and six weeks after initial STZ injection. Before collecting baseline data, mice were acclimated to the testing areas, mesh table, and thermal testing apparatus for 30 min on two occasions, separated by 24 h. Mice were also acclimated to the testing areas, mesh table, and thermal testing apparatus for 30 min each before every testing session. Mechanical sensitivity was measured using Von Frey monofilaments applied to the plantar surface of the hind paw following the “up-down” method (Chaplan et al., 1994). If there was no response to a filament, the next higher filament was used until a response was elicited. The next lower filament was applied when a positive response was observed. This pattern was followed for four trials following a positive response. Thermal sensitivity was determined using a glass thermal testing apparatus. A 4.2 V radiant heat source was applied to the hind paw’s plantar surface, and the withdrawal latency was recorded three times.

### IENFD

Six weeks after initial STZ injection, mice were euthanized using isoflurane overdose in compliance with the University of Kansas Medical Center’s Institutional Animal Care and Use Committee. Hind paws were immediately collected, immersed in Zamboni’s fixative overnight, and rinsed overnight in 1 % PBS. The footpads were then removed and immersed in 30 % sucrose in PBS overnight. The tissue was cryopreserved in Optimal Cutting Temperature Compound (Sekura Tissue-Tek). Thirty-micron sections were blocked for two hours at room temperature in Superblock (ThermoFisher), 1.5 % Normal Donkey Serum, 0.5 % Porcine Gelatin, and 0.5 % Triton X-100 (Sigma). Slides were incubated overnight with rabbit α-PGP9.5 (1:1500, ProteinTech, Cat #14730–1-AP) diluted in blocking solution. Slides were then incubated with AlexaFluor-555 tagged donkey-α-rabbit secondary antibody (1:1000; Molecular Probes) for one hour and imaged with a Nikon Eclipse 90i microscope using a 20X objective. Nikon Elements software was used to measure the length of the dermal-epidermal junction. IENFD was quantified as the number of fibers crossing the dermal-epidermal junction and expressed as the number of fibers per millimeter. Nine images were taken per mouse from three tissue sections (three images per section), and the average IENFD for each mouse was used for statistical analyses.

### Dermal macrophage measurement

Thirty-micron footpad sections were collected and blocked as described previously, then incubated overnight with rabbit α-Iba1 (1:500, Wako, Cat #019–19741) diluted in blocking solution. Slides were then incubated with AlexaFluor-555-tagged donkey-α-rabbit secondary antibody (1:1000; Molecular Probes) for one hour and imaged using a Nikon Eclipse 90i microscope with a 10X objective. Serial images were stitched together using Nikon Elements software to create a final image that encompassed the entire length of the footpad section. All images were taken at the same exposure. ImageJ was then used to measure the intensity of the area of interest, which included the epidermal-dermal junction and 500 μm below the junction. The intensities of three regions lacking macrophage staining within the area of interest were measured, averaged, and subtracted from the overall intensity to account for background. Three tissue sections were measured per mouse, and the average Iba1 intensity for each mouse was used for statistical analyses. Although Iba1 marks both macrophages and Langerhans cells, the latter are restricted primarily to the epidermis (Neagu et al., 2022). Because our measurements were taken below the epidermis, the contribution of Langerhans cells to the observed Iba1 signal is expected to be minimal.

### Serum cytokine bead array

Blood was collected at sacrifice using cardiac puncture, allowed to clot for one hour at room temperature, and centrifuged at 1,500 × g for 15 min. The resulting serum supernatant was collected and stored at −80 °C. A panel of 13 cytokines was measured using the LEGENDplex Mouse Inflammation Panel (Cat #740446). The serum was diluted 1:8 in Assay Buffer and Matrix C, following the kit’s directions to minimize matrix effect. The assay was performed according to the manufacturer’s protocol and read on the Attune NxT Flow Cytometer (ThermoFisher). Data was analyzed using LEGENDplex Qognit software. Sample concentrations below the level of detection were extrapolated from the standard curve generated by the Qognit software and included in the analysis. Cytokines with ≥ 50 % of sample values below the limit of detection (LOD) were excluded from analysis. Excluded cytokines included IFN-γ, TNF-α, CCL2 (MCP-1), IL-12p70, IL-1β, IL-10, IL-6, IL-27, IL-17A, IFN-β, and GM-CSF. No samples were above the upper limit of detection for any cytokine.

### Statistical analysis

Statistical analysis was performed using R version 4.2.0. For data collected at multiple time points, preliminary mixed-design ANOVAs with time as a within-subject factor and sex, genotype, and treatment as between-subject factors were run to test for sex effects. If there was no significant effect of sex, it was excluded as a factor, and the mixed-design ANOVA was rerun. One-way ANOVAs with Tukey’s Honest Significant Difference (HSD) were used to determine differences between groups at individual time points. Area under the curve (AUC) was analyzed using three-way ANOVAs for sex, genotype, and treatment with Tukey’s HSD post hoc test. IENFD, Iba1 intensity, and serum cytokines were analyzed using two-way ANOVAs for genotype and treatment with Tukey’s HSD post hoc test. IENFD percent change from control was analyzed using an unpaired Student *t*-test. Pearson correlation was used to analyze the linear relationship between variables. All data are presented as mean +/− standard error of the mean.

## Results

### STZ treatment induces hyperglycemia in both sexes and weight loss in males, independent of TLR4−/− genotype

Blood glucose was measured at three days (following either the first or second dose of STZ, if required) and at three and six weeks after initial STZ injection. STZ-treated females had significantly elevated blood glucose compared to their respective controls at all time points, independent of genotype (Fig. 1B; 3 days: WT *p* < 0.001, TLR4−/− *p* = 0.002; *p* < 0.001 for all remaining comparisons). STZ-treated males also had significantly elevated blood glucose at all time points independent of genotype (Fig. 1C; *p* < 0.001 for all comparisons), indicating that STZ induced similar levels of hyperglycemia in both wild-type and TLR4−/− mice. However, male mice developed more severe STZ-induced hyperglycemia than female mice across both genotypes (Fig. 1D–E). This was significant at three (*p* = 0.006) and six (*p* < 0.001) weeks for wild-type mice and at all time points (*p* < 0.001 for all comparisons) for TLR4−/− mice.

Body weight was recorded at baseline and at three and six weeks following STZ injection. STZ-treated females of both strains did not experience a significant change in body weight at any time point compared to their vehicle-treated controls (Fig. 1F). In contrast, STZ-treated male mice had a significantly lower body weight than controls at three (WT *p* < 0.001; TLR4−/− *p* = 0.002) and six weeks (*p* < 0.001 for all comparisons) post-STZ injection (Fig. 1G). Additionally, STZ-treated wild-type male mice had a significantly lower body weight than STZ-treated TLR4−/− male mice at six weeks (*p* = 0.027).

These findings indicate that STZ induces hyperglycemia in wild-type and TLR4−/− mice. However, male mice develop more severe hyperglycemia in response to STZ than female mice in both strains. STZ also appears to only induce weight loss in male mice, and TLR4−/− may provide a small amount of protection against STZ-induced weight loss in males.

### TLR4 deletion mitigates STZ-induced thermal hyposensitivity, and STZ does not alter mechanical sensitivity in either genotype

To assess peripheral sensation, we measured mechanical sensitivity using Von Frey filaments and thermal sensitivity using a radiant heat assay at two, four, and six weeks post-STZ injection.

**Fig. 2.**
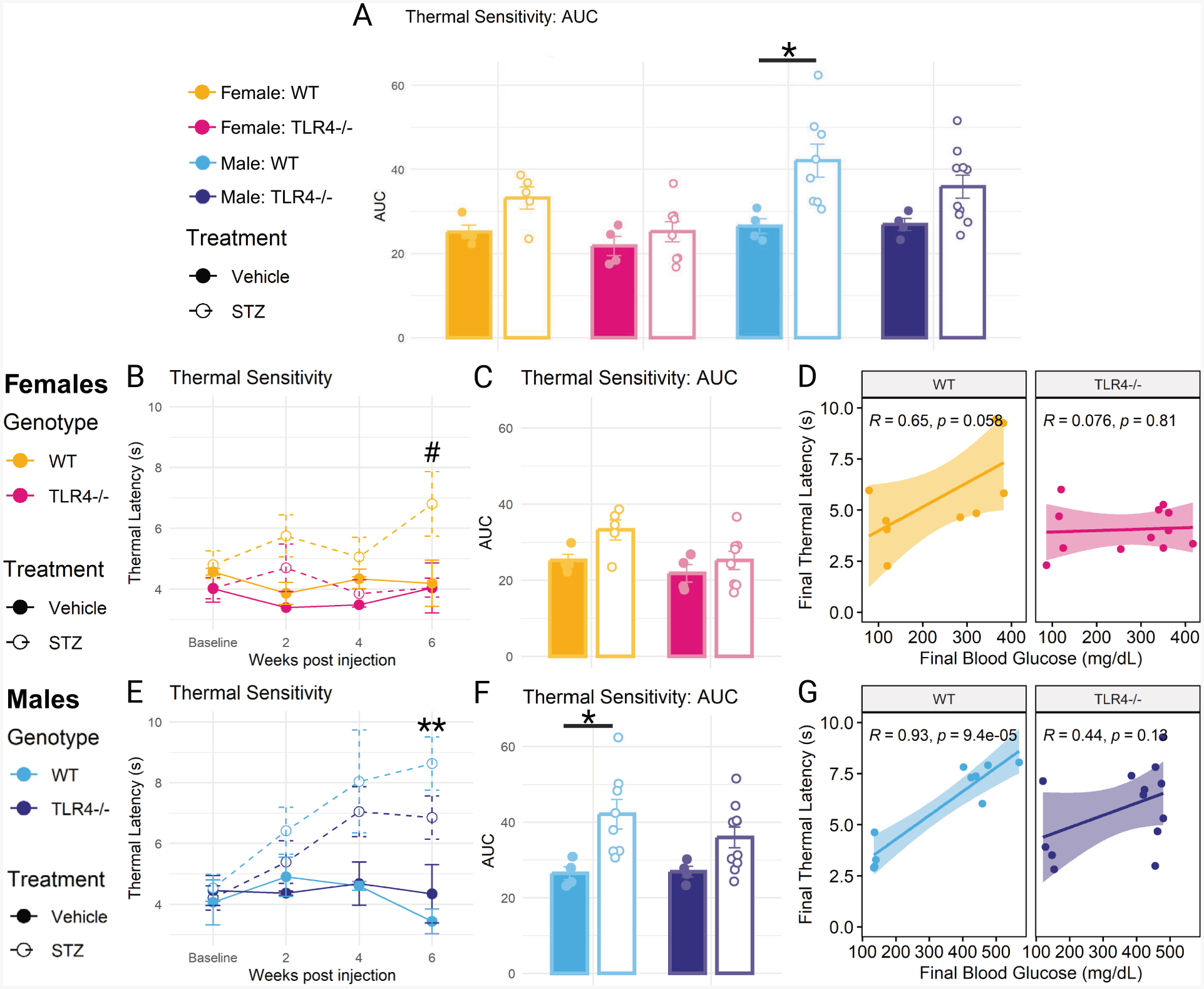
Thermal sensitivity in the hind paw. (A) Area under the curve (AUC) analysis of thermal latency in male and female wild-type (WT) and TLR4−/− mice treated with STZ or vehicle. (B) Thermal latency in seconds at baseline, 2, 4, and 6 weeks in STZ- or vehicle-treated WT and TLR4−/− females. (C) AUC analysis of thermal latency in STZ- or vehicle-treated WT and TLR4−/− females. (D) Correlation between final thermal latency and blood glucose at 6 weeks in WT and TLR4−/− females. (E) Thermal latency in seconds at baseline, 2, 4, and 6 weeks in STZ- or vehicle-treated WT and TLR4−/− males. (F) AUC analysis of thermal latency in STZ- or vehicle-treated WT and TLR4−/− males. (G) Correlation between final thermal latency and blood glucose at 6 weeks in WT and TLR4−/− males. AUC analyzed using three-way ANOVA with Tukey’s HSD post-hoc test (* p ≤ 0.05). n = 4–10. Time course analyzed using one-way ANOVA with Tukey’s HSD post-hoc test at each time-point (# p ≤ 0.05 STZ-treated WT vs STZ-treated TLR4−/−, ** p ≤ 0.01 STZ-treated WT vs vehicle-treated WT). n = 4–10. Correlation analyzed using Pearson correlation coefficient, plotted with linear regression line and 95 % confidence interval. n = 9–14. Data are represented as means ± SEM.

Area under the curve (AUC) analysis comparing thermal sensitivity across all groups (Fig. 2A) showed a significant difference in thermal latency between STZ-treated wild-type male mice and their vehicle-treated controls, with STZ-treated mice developing thermal hyposensitivity (*p* = 0.029). This difference was not present in TLR4−/− male mice. Additionally, there was significant main effect of sex (*F*(1,39) = 15.37, *p* < 0.001), with STZ-treated female mice developing significantly less thermal hyposensitivity than STZ-treated male mice (*p* = 0.003). Wild-type and TLR4−/− STZ-treated female mice were not significantly different than their respective controls on AUC analysis.

Comparison of thermal sensitivity in female mice across time points (Fig. 2B) revealed a difference between wild-type and TLR4−/− STZ-treated groups at six weeks (*p* = 0.034), although neither differed from their respective control. No difference between any group was found on AUC analysis (Fig. 2C). A nearly significant correlation was found between final thermal latency and final blood glucose in wild-type female mice (*R* = 0.65, *p* = 0.058), with higher blood glucose correlating with greater thermal hyposensitivity (Fig. 2D). This was not present for TLR4−/− female mice.

STZ-treated wild-type males developed significant thermal hyposensitivity compared to their vehicle-treated controls by six weeks (*p* = 0.004), while STZ-treated TLR4−/− males did not develop significant thermal hyposensitivity at any time point (Fig. 2E). AUC analysis showed a significant effect of STZ treatment in wild-type males (*p* = 0.029), but not in TLR4−/− males (Fig. 2F). Additionally, there was a strong correlation between final thermal latency and final blood glucose in wild-type males (*R* = 0.93, *p* < 0.0001) that was not present in TLR4−/− males (Fig. 2G).

**Fig. 3.**
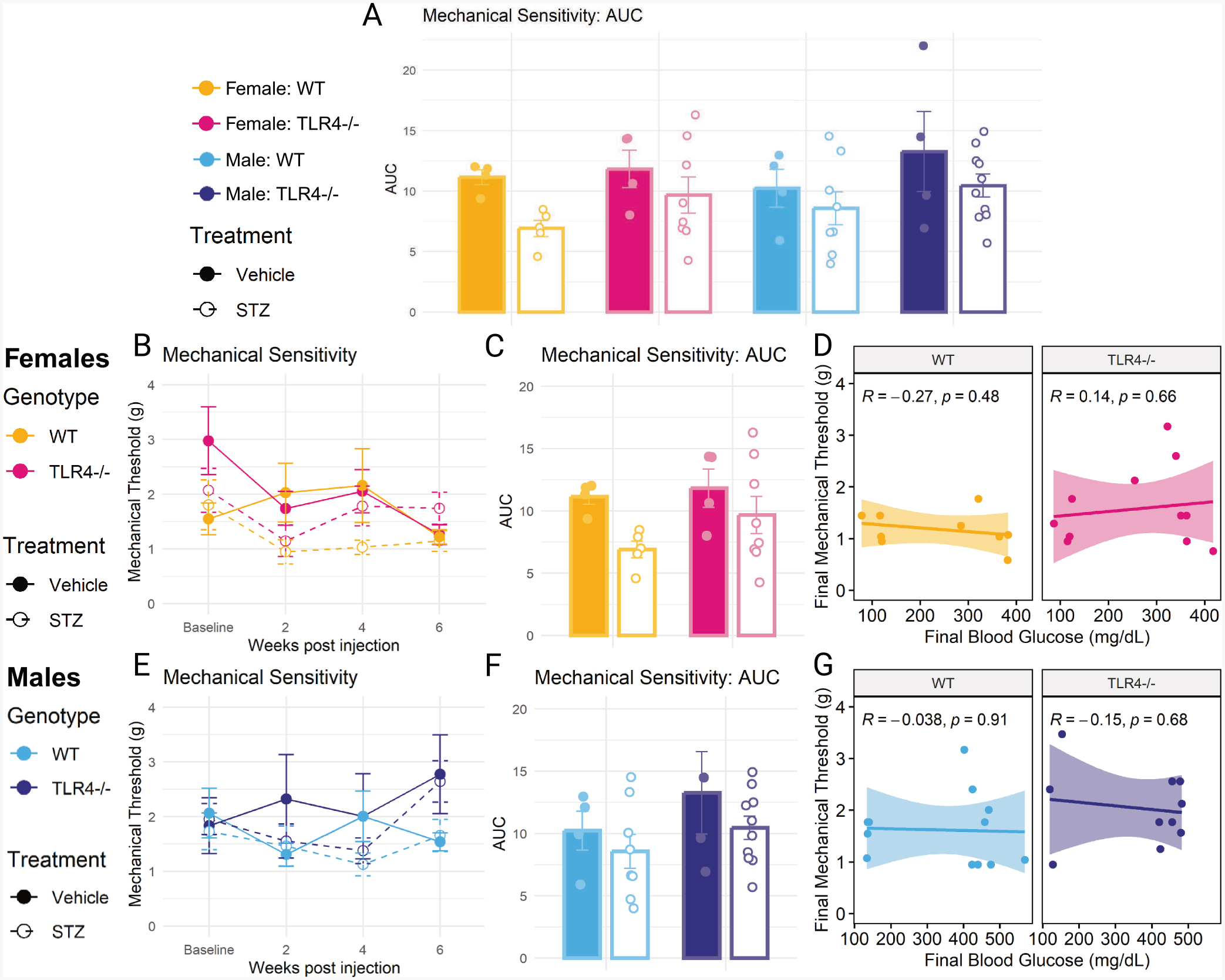
Mechanical sensitivity in the hind paw. (A) Area under the curve (AUC) analysis of mechanical threshold in male and female wild-type (WT) and TLR4−/− mice treated with STZ or vehicle. (B) Mechanical threshold in grams at baseline, 2, 4, and 6 weeks in STZ- or vehicle-treated WT and TLR4−/− females. (C) AUC analysis of mechanical threshold in STZ- or vehicle-treated WT and TLR4−/− females. (D) Correlation between final mechanical threshold and blood glucose at 6 weeks in WT and TLR4−/− females. (E) Mechanical threshold in seconds at baseline, 2, 4, and 6 weeks in STZ- or vehicle-treated WT and TLR4−/− males. (F) AUC analysis of mechanical threshold in STZ- or vehicle-treated WT and TLR4−/− males. (G) Correlation between final mechanical threshold and blood glucose at 6 weeks in WT and TLR4−/− males. AUC analyzed using three-way ANOVA with Tukey’s HSD post-hoc test. n = 4–10. Time course analyzed using one-way ANOVA with Tukey’s HSD post-hoc test at each time-point. n = 4–10. Correlation analyzed using Pearson correlation coefficient, plotted with linear regression line and 95 % confidence interval. n = 9–14. Data are represented as means ± SEM.

AUC analysis of mechanical data (Fig. 3 A–G) revealed a significant main effect of STZ treatment (*F*(1,39) = 5.620, *p* = 0.023), with STZ treatment lowering mechanical thresholds. Analysis of the data across time showed a significant main effect of genotype (*F*(1,43) = 4.939, *p* = 0.032), with TLR4−/− mice having a higher mechanical threshold. However, no individual groups were significantly different from each other at any time point or on AUC analysis, and no correlations with final blood glucose were found.

### STZ-induced epidermal nerve fiber loss is not observed in females or TLR4−/− males

**Fig. 4.**
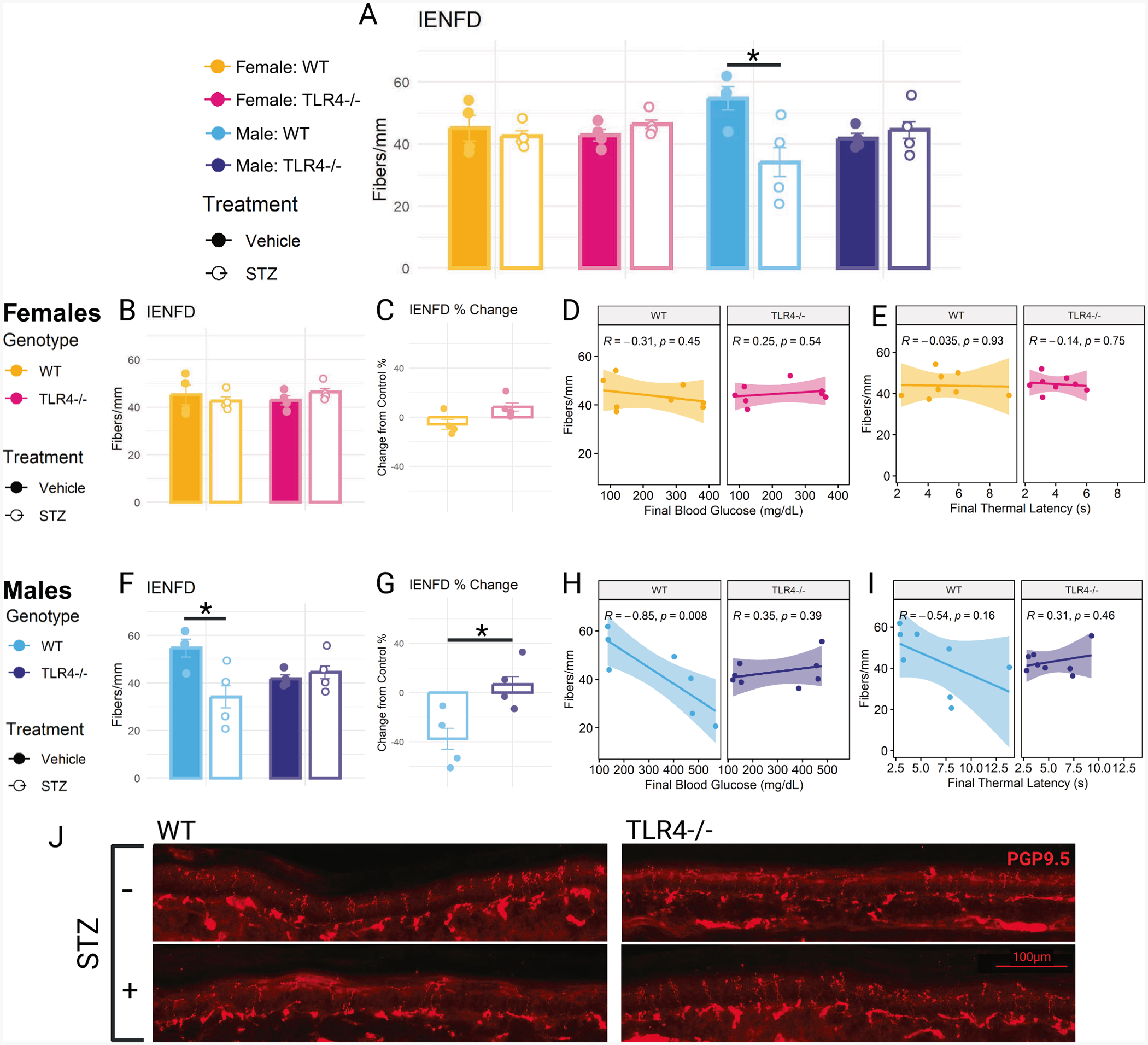
Intraepidermal nerve fiber density (IENFD) in the hind paw. (A) IENFD, measured as the number of fibers crossing the dermal-epidermal junction per mm, six weeks after initial injection in STZ- or vehicle-treated wild-type (WT) and TLR4−/− female and male mice (B) IENFD in STZ- or vehicle-treated WT and TLR4−/− females. (C) Percent change of IENFD in female STZ-treated WT and TLR4−/− mice compared to their respective vehicle-treated controls. (D) Correlation between IENFD and final blood glucose in WT and TLR4−/− female mice. (E) Correlation between IENFD and final thermal latency in WT and TLR4−/− female mice. (F) IENFD in STZ- or vehicle-treated WT and TLR4−/− males. (G) Percent change of IENFD in male STZ-treated WT and TLR4−/− mice compared to their respective vehicle-treated controls. (H) Correlation between IENFD and final blood glucose in WT and TLR4−/− male mice. (I) Correlation between IENFD and final thermal latency in WT and TLR4−/− male mice. (J) Representative 20X images of PGP9.5 + nerve fibers crossing the dermal-epidermal junction in the hind paw from STZ- and vehicle-treated WT and TLR4−/− male mice. IENFD analyzed using two-way ANOVA with Tukey’s HSD post-hoc test (* p ≤ 0.05). n = 4. Percent change analyzed using unpaired student *t*-test (* p ≤ 0.05). n = 4. Correlation analyzed using Pearson correlation coefficient, plotted with linear regression line and 95 % confidence interval. n = 8. Data are represented as means ± SEM.

Reduced IENFD is a hallmark of diabetic neuropathy (Lauria et al., 2010). After six weeks, only STZ-treated wild-type males experienced a significant decrease in IENFD (Fig. 4A). STZ-treated females of both genotypes did not experience a decrease in IENFD (Fig. 4B–C). Additionally, no correlations between IENFD and final blood glucose or thermal latency were observed for females (Fig. 4D–E).

Unlike the females, STZ-treated wild-type males experienced a significant decrease in IENFD (Fig. 4F; *p* = 0.028), losing approximately 40 % of their fibers compared to their control (Fig. 4G). STZ-treated TLR4−/− male mice were protected from a decrease in IENFD. Their baseline IENFD appeared lower, although not significantly, than that of wild-type mice. Compared to their vehicle-treated control, STZ-treated TLR4−/− males had a slightly higher IENFD and had a significantly different percent change from control compared to wild-type mice (Welch’s *t*(5.816) = −2.822, *p* = 0.031). In wild-type males, IENFD was strongly negatively correlated with final blood glucose (Fig. 4H; *R* = −0.85, *p* = 0.008) and showed a nonsignificant trend towards a negative correlation with final thermal latency (Fig. 4I; *R* = −0.54, *p* = 0.16). These correlations were not present for TLR4−/− males. Representative images from male mice show a noticeable decrease in nerve fibers crossing the dermal-epidermal junction in STZ-treated wild-type mice that is not observed in TLR4−/− mice (Fig. 4J).

### Females and TLR4−/− males do not exhibit STZ-Induced dermal macrophage aggregation

**Fig. 5.**
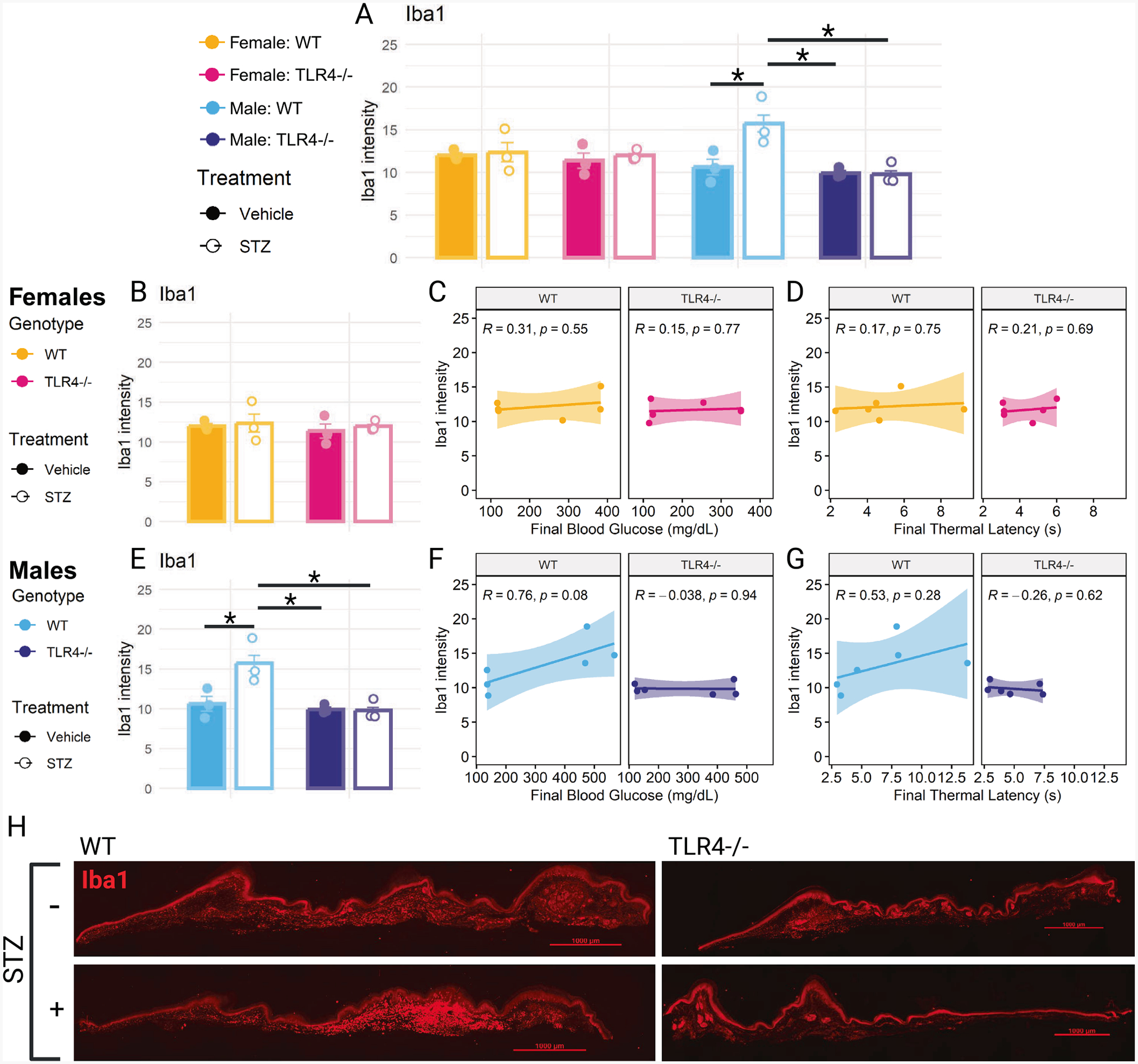
Iba1 + macrophages in the hind paw. (A) Iba1 intensity measured in the dermis of full cross-sections of the hind paw in STZ- or vehicle-treated wild-type (WT) and TLR4−/− female and male mice. (B) Hind paw Iba1 intensity in STZ- or vehicle-treated WT and TLR4−/− female mice. (C) Correlation between Iba1 intensity and final blood glucose in WT and TLR4−/− female mice. (D) Correlation between Iba1 intensity and final thermal latency in WT and TLR4−/− female mice. (E) Hind paw Iba1 intensity in STZ- or vehicle-treated WT and TLR4−/− male mice. (F) Correlation between Iba1 intensity and final blood glucose in WT and TLR4−/− male mice. (G) Correlation between Iba1 intensity and final thermal latency in WT and TLR4−/− male mice. (H) Representative images of Iba1 + macrophages in full hind paw cross-sections from STZ- and vehicle-treated WT and TLR4−/− male mice. Iba1 intensity analyzed using two-way ANOVA with Tukey’s HSD post-hoc test (* p ≤ 0.05). n = 3. Correlation analyzed using Pearson correlation coefficient, plotted with linear regression line and 95 % confidence interval. n = 6. Data are represented as means ± SEM.

Iba1 was used to examine macrophages in the hind paw dermis. An increase in Iba1 intensity was observed only in wild-type male mice following STZ treatment (Fig. 5A). STZ-treated females did not experience a change in Iba1 intensity in either genotype (Fig. 5B) and Iba1 intensity did not correlate with final blood glucose (Fig. 5C) or thermal latency (Fig. 5D). In contrast, STZ-treated wild-type males had significantly higher Iba1 intensity compared to their vehicle-treated controls (*p* = 0.036) and STZ-treated TLR4−/− mice (Fig. 5E*; p* = 0.016). Iba1 intensity had a strong and nearly significant correlation with final blood glucose in wild-type males (Fig. 5F; *R* = 0.76, *p* = 0.08). It also had a moderate, but non-significant correlation with final thermal latency in wild-type males (Fig. 5G; *R* = 0.53, *p* = 0.28). These correlations were not present for TLR4−/− males. Iba1 intensity did not correlate with IENFD for any sex or genotype. Imaging revealed sporadic aggregates of Iba1 + macrophages in STZ-treated wild-type male mice (Fig. 5H).

### Serum IL-23 and IL-1α are not altered by STZ treatment or TLR4−/−

**Fig. 6.**
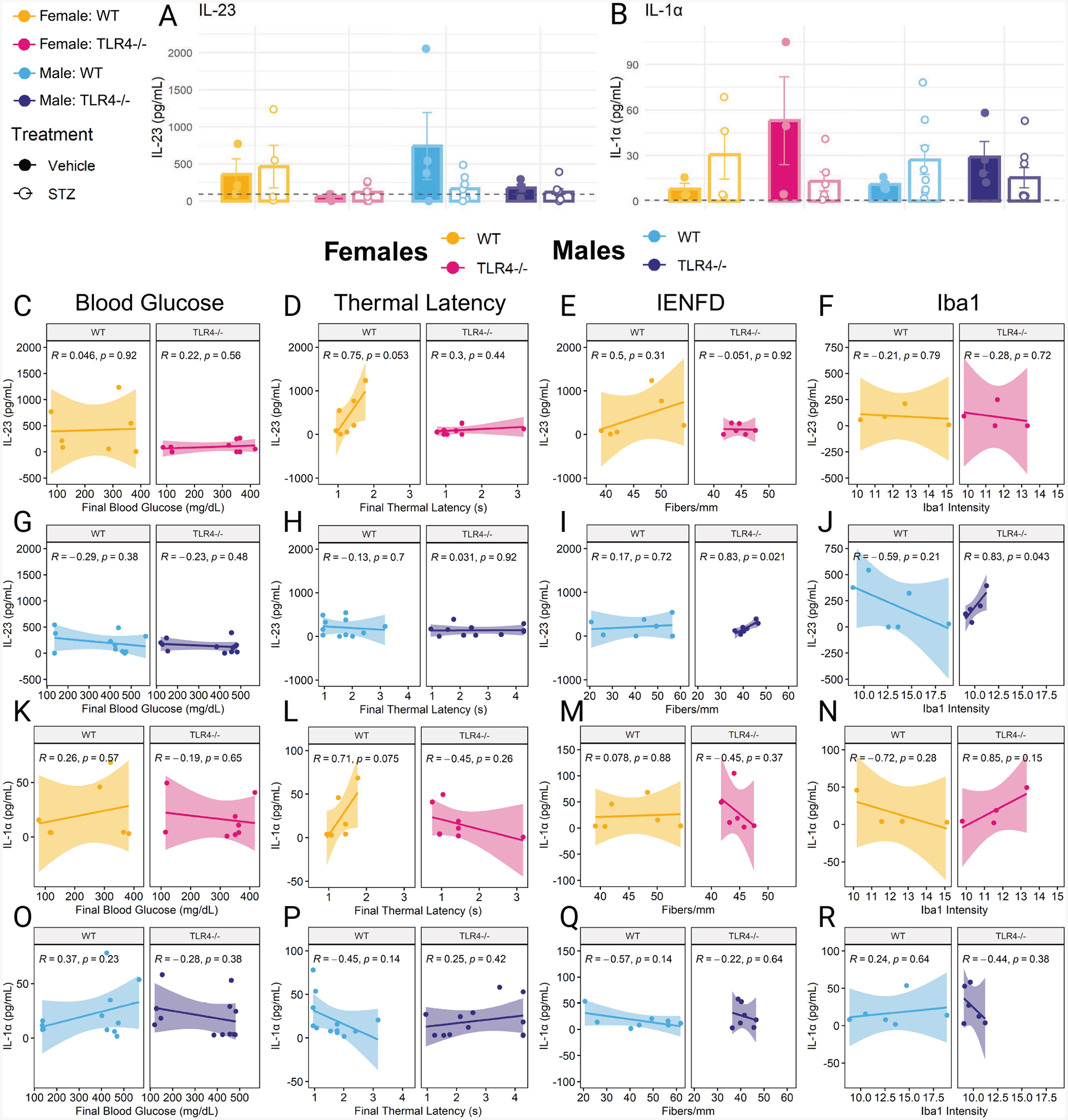
Serum IL-23 (A) and IL-1α (B) at six weeks post-initial STZ or vehicle injection measured using cytokine bead array in wild-type (WT) and TLR4−/− female and male mice. Gray dashed lines indicate the LOD for IL-23 (92 pg/mL) and IL-1α (0.58 pg/mL). Correlation between IL and 23 and final blood glucose, final thermal latency, IENFD, and Iba1 intensity in females (C-F) and males (G-J). Correlation between IL and 1α and final blood glucose, final thermal latency, IENFD, and Iba1 intensity in females (K-N) and males (O-R). Serum cytokines analyzed using two-way ANOVA with Tukey’s HSD post-hoc test. n = 3–8. Correlation analyzed using Pearson correlation coefficient, plotted with linear regression line and 95 % confidence interval. n = 4–12. Data are represented as means ± SEM.

To assess systemic inflammation, we measured circulating cytokine levels at the study’s conclusion. Although 13 cytokines were measured, only IL-23 and IL-1α had sufficient samples above the limit of detection to be considered analyzable. IL-23 levels appeared to be higher in wild-type mice compared to TLR4−/− mice, but this was not significant for either sex (Fig. 6A). IL-1α levels appeared to be trending towards being higher in wild-type STZ-treated mice of both sexes compared to their vehicle-treated controls (Fig. 6B). Interestingly, IL-1α appeared higher in vehicle-treated TLR4−/− animals compared to STZ-treated animals. However, none of these groups were significantly different. Correlations between final blood glucose, thermal latency, IENFD, and Iba1 intensity were examined in relation to IL-23 (Fig. 6C–J) and IL-1α (Fig. 6 K–R) in males and females. IL-23 significantly correlated with IENFD (*R* = 0.83, *p* = 0.021) and Iba1 intensity (*R* = 0.83, *p* = 0.043) in male TLR4−/− mice. However, it should be noted that the ranges of IENFD and Iba1 intensity values measured in this group were narrow. No significant correlations were found for IL-1α.

## Discussion

Neuropathy is a common chronic complication of diabetes, and its prevalence continues to rise globally. The innate immune receptor TLR4 has been implicated in neuropathic sensory symptoms across various neuropathy models, including chronic constriction injury (Bettoni et al., 2008; Liu et al., 2016; Piao et al., 2018; Wu et al., 2010), spared nerve injury (Szabo-Pardi et al., 2021), spinal nerve transection (Tanga et al., 2005), autoimmune peripheral neuropathy (Oladiran et al., 2021), chemotherapy-induced peripheral neuropathy (Li et al., 2014), and high-fat-diet-induced metabolic neuropathy (Elzinga et al., 2019). However, the role of TLR4 in the progression of hyperglycemia-related diabetic neuropathy and nerve fiber density loss in the skin has not been investigated.

To assess the impact of TLR4 on sensory dysfunction, nerve fiber loss, and inflammation in diabetes, we induced diabetes in wild-type and TLR4−/− mice using STZ. Over a period of six weeks, we monitored blood glucose levels, body weight, sensory function, IENFD, dermal macrophages, and serum cytokines. Our results show that, in males, global TLR4 gene deletion protects against thermal hyposensitivity, nerve fiber loss, and macrophage accumulation in the hind paw. Female mice of both strains developed a milder diabetic phenotype, including less severe hyperglycemia, no significant weight loss, preserved epidermal fiber density, and no increase in dermal macrophages. Thermal hyposensitivity was also less pronounced in females, although STZ-treated wild-type females exhibited significantly higher thermal latency than STZ-treated TLR4−/− females by six weeks. Although the lack of an outward neuropathic phenotype makes it difficult to discern the effect of TLR4−/− in females, it highlights the need for a better understanding of the complex sex differences in diabetic models and neuropathy pathogenesis.

Consistent with previous findings, STZ-treated wild-type male mice developed thermal hyposensitivity (Enders et al., 2022). TLR4 deletion appeared to mitigate this effect. This was supported by a strong positive correlation between final blood glucose and final thermal latency in wild-type males that was absent in TLR4−/− males, suggesting that TLR4−/− mice were protected from thermal hyposensitivity associated with hyperglycemia. These results align with prior findings that TLR2/4 global null mutant mice on a high-fat diet developed thermal hyposensitivity later than wild-type mice (Elzinga et al., 2019). TLR4 gene deletion also reduces sensory symptoms in direct nerve injury (Bettoni et al., 2008; Liu et al., 2016; Piao et al., 2018; Szabo-Pardi et al., 2021; Tanga et al., 2005; Wu et al., 2010) and chemotherapy-induced neuropathy models (Li et al., 2014). Here, for the first time, we confirm TLR4′s protective role in diabetic neuropathy in male mice.

TLR4 deletion was also protective in preserving nerve fiber density in the epidermis in males. STZ-treated wild-type male mice exhibited a ~ 40 % reduction in IENFD compared to their vehicle-treated control, whereas STZ-treated TLR4−/− male mice displayed a slight increase. Additionally, a strong negative correlation between IENFD and final blood glucose was found for wild-type male mice, but no correlation was found for TLR4−/− male mice. Though not statistically significant, TLR4−/− mice had lower baseline IENFD than wild-type mice. Since TLR4 plays a role in nerve fiber regeneration (Wu et al., 2013) and neural precursor cell development (Sanchez-Petidier et al., 2022), its absence may impair neuronal development and regeneration, leading to reduced baseline IENFD.

Additionally, TLR4 deletion prevented an STZ-induced increase in dermal Iba1 + intensity in male mice, indicating reduced activation of dermal macrophages. Increased dermal Iba1 + and CD68 + macrophage densities have been reported in type 2 diabetic neuropathy patients (Shepherd et al., 2018; Üçeyler et al., 2017), particularly in painful neuropathy cases (Gylfadottir et al., 2022), though contrasting studies show reduced Iba1 + macrophages in type 1 diabetic patients with neuropathy (Hu et al., 2023). This discrepancy may stem from differences in disease duration and pathophysiological differences between type 1 and type 2 diabetes. To differentiate whether macrophages in the hind paw are infiltrating versus proliferating, a future direction could be to assess for markers such as CCR2.

Importantly, TLR4−/− did not directly improve STZ-induced hyperglycemia. This indicates that the outcomes we observed were not due to TLR4−/− attenuating the development of diabetes. STZ induced more severe hyperglycemia in male mice, a well-documented phenomenon in C57BL/6 (Chandramouli et al., 2018; Kim et al., 2020) and outbred ICR (Kim et al., 2023a) mice, attributed to estrogen’s protective effect on β-cell function by promoting misfolded proinsulin degradation (Xu et al., 2018). This is supported by the fact that female mice are more resistant to STZ-induced diabetes and require higher doses to develop hyperglycemia (Saadane et al., 2020). This was also true in our study, as more female mice required repeat doses of STZ than male mice. This carries clinical relevance, as estrogen is protective against the development of diabetes, and lower estrogen levels increase diabetes risk in premenopausal and ovariectomized women (Malacara et al., 1997; Park et al., 2017). However, females with diabetes report greater neuropathic pain intensity than males, despite nerve injury being more common in males (Abraham et al., 2018).

Consistent with this, our study reflected greater nerve injury observed in males. Previous pre-clinical studies report greater IENFD loss in males in obesity-related diabetic *ob/ob* models (O’Brien et al., 2016), though not in high-fat diet models (Elzinga et al., 2021), possibly due to differing measurement time points. Limited research exists on sex-specific STZ effects on IENFD, and our findings highlight male-specific vulnerability in the STZ model. Although our IENFD findings align with clinical observation, our study did not capture the increased pain intensity reported in females. It remains unclear whether this discrepancy reflects limitations of our behavioral assays, the subjective nature of pain, or an inability of the STZ model to induce neuropathic pain in females within the observed time course. However, our findings emphasize sex-specific effects of the STZ model in diabetic neuropathy. Given that female sex is a risk factor for painful diabetic neuropathy (Elliott et al., 2024), inclusion of females in pre-clinical research remains essential. New methods are emerging showing that intravenous STZ injection may be a promising way to induce neuropathy in females and should be considered as a future method (Hagenimana et al., 2025).

Although the lack of a neuropathic phenotype makes it difficult to draw conclusions about the impact of TLR4−/− on diabetic neuropathy in females, our findings indicate a protective role in male mice. This contrasts with findings in a sciatic nerve injury model, where TLR4 gene deletion in sensory nerves was protective only in females (Szabo-Pardi et al., 2021). The sex-specific role of TLR4 in pain development remains complex, as previous research also indicates that TLR4 activation in the spinal cord induces neuropathic hypersensitivity in males but not in females (Sorge et al., 2011). Further research is needed to fully understand TLR4′s sex-specific role in diabetic neuropathy.

In this study, we did not detect changes in IL-23 or IL-1α in response to STZ treatment or TLR4 deletion. While TLR4 is typically implicated in promoting the production of proinflammatory cytokines such as IL-23 and IL-1α (Eltom et al., 2014; Kang et al., 2018), this effect may not have been evident here because the chronic diabetic state did not elicit a robust increase in circulating cytokines, leading to high variability across groups. Although no cytokines or downstream mediators are entirely specific to TLR4 signaling, more sensitive assays targeting TNFα or IL-1β could help clarify the impact of TLR4 deletion on systemic inflammation in the context of STZ-induced diabetes.

A limitation of this study is the duration of the assessment related to diabetes-related consequences. A longer time course may have provided greater insight into the progression of neuropathy in female mice in the absence of TLR4. However, prolonged STZ-induced diabetes in male mice increases lethality beyond six weeks, somewhat limiting the feasibility of extended observation. Additionally, the TLR4 gene deletion was global, affecting TLR4 expression in multiple cell types. Relevant to this study, TLR4 is expressed on macrophages and sensory neurons, promoting the release of inflammatory cytokines and chemokines, which in turn recruit additional immune cells (Kim et al., 2023b; Wei et al., 2023). Diabetes can trigger macrophage dysregulation in the dermis, which is thought to contribute to impaired wound healing (Domoto et al., 2021; Gylfadottir et al., 2022; Sharifiaghdam et al., 2022). Additionally, there is evidence that dermal macrophages are involved in modulating pain sensitivity by releasing nerve growth factor (Tanaka et al., 2023). Additional studies are needed to differentiate immune- and neural-specific TLR4-related effects.

These findings demonstrate that TLR4 deletion does not impact diabetes-induced hyperglycemia, but at least in part mitigates thermal hyposensitivity, peripheral nerve fiber loss, and dermal macrophage aggregation in male mice. Male mice exhibited more severe hyperglycemia and resulting neuropathy than female mice in this model, making the protective effects of TLR4 deletion more apparent in males. These results suggest that TLR4 contributes to the pathophysiology of diabetic neuropathy, sensory deficits, and nerve degeneration, making it an additional therapeutic target.

## Figures and Tables

**Table 1:** Final number of mice per group before and after exclusion. Group size variability is due to the availability of TLR4−/− mice (colony was maintained in-house) and differences in susceptibility to STZ-induced diabetes between males and females.

		N (N before exclusion)		
		Male	Female	Total
Wild-type	Vehicle	4	4	**8**
	STZ	8	5 (8)*	**13 (16)**
TLR4−/−	Vehicle	4	4	**8**
	STZ	10 (13)**	8 (14)*	**18 (27)**

* Mice were excluded because they did not reach the blood glucose level required to be considered diabetic.

** Mice were excluded because they reached a humane endpoint and were euthanized before study completion.

## Abbreviations:

DPN: diabetic peripheral neuropathy
TLR4: toll-like receptor 4
TLR4−/−: toll-like receptor 4 knockout
IENFD: intraepidermal nerve fiber density
TLRs: toll-like receptors
LPS: lipopolysaccharide
STZ: streptozotocin
WT: wild-type
LOD: limit of detection
